# Sequence-Dependent Interaction of the Human Papillomavirus E2 Protein with the DNA Elements on Its DNA Replication Origin

**DOI:** 10.3390/ijms24076555

**Published:** 2023-03-31

**Authors:** Gulden Yilmaz, Esther E. Biswas-Fiss, Subhasis B. Biswas

**Affiliations:** 1 Department of Molecular Biology, Rowan University, Stratford, NJ 08084, USA; 2 Department of Medical and Molecular Sciences, University of Delaware, College of Health Sciences, Newark, DE 19716, USA; 3 Ammon Pinizzotto Biopharmaceutical Innovation Center, 590 Avenue 1743, Newark, DE 19713, USA

**Keywords:** human papillomavirus, E2 binding site, electrophoretic mobility shift assay, dissociation constant, Atomic Force Microscopy (AFM)

## Abstract

The human papillomavirus (HPV) E2 protein is essential for regulating the initiation of viral DNA replication as well as the regulation of transcription of certain HPV-encoded genes. Its ability to recognize and bind to its four recognition sequences in the viral origin is a key step in the initiation of HPV DNA replication. Thus, understanding the mechanism of DNA binding by E2 protein and the unique roles played by individual DNA sequence elements of the replication origin is essential. We have purified the recombinant full-length HPV type 11 E2 protein. Quantitative DNA binding analysis indicated E2 protein bound all four DNA binding sites with reasonably high affinities but with distinct preferences. It bound its cognate binding sites 1, 2, and 4 with higher affinities, but bound binding site 3 with lower affinity. Analysis of binding to these sites unraveled multiple sequence elements that appeared to influence E2 binding affinity and target discrimination, including the sequence of spacer region, flanking sequences, and proximity of E2 binding sites. Thermodynamic analysis indicated hydrophobic interaction in the protein-DNA complex formation. Our studies indicate a large multi-protein complex formation on the HPV-origin DNA, likely due to reasonably high binding affinities as well as intrinsic oligomerization propensity of E2 dimers.

## 1. Introduction

Human papillomavirus (HPV) is the most common sexually transmitted disease in the United States, with ~80% of all women infected by the age of 50 [1]. The most common group of HPVs consists of the alpha papillomaviruses, which are further classified as low-risk and high-risk based on their ability to induce cancer [2]. Low-risk or non-oncogenic HPVs, which include common HPV-6 and HPV-11 strains, are correlated with benign lesions, such as low-grade cervical abnormalities, and 90% of all anogenital wart cases [3]. These low-risk HPV types also commonly occur in recurrent respiratory papillomatosis (RRP), which is a life-threatening disease caused by the recurrence of warts or papillomas present in the upper respiratory tract [4].

**Figure 1:**
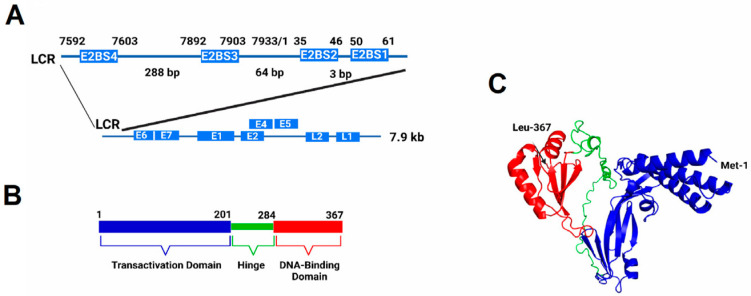
Structure of E2 protein, HPV-11 genome, and its predicted crystal structure. (**A**) The chromosomal location and structure of the DNA replication origin in the HPV-11 genome. The enlargement of the LCR indicates the location of the four E2 binding sites (E2 BS1-4) (not drawn to scale). (**B**) Structure of the HPV-11 full-length E2 protein indicating the N-terminal transactivation, hinge, and C-terminal DNA-binding domains (drawn to scale). (**C**) A homology-based model of full-length E2 protein created using the Robetta Structure Prediction software (3 March 2023) at the University of Washington depicting structures of the transactivation and DNA-binding domains. The first and last amino acids covered in the model are annotated by MET-1 and LEU-367, respectively.

HPV is a double-stranded DNA tumor virus, and more than 100 HPV types have been identified [5]. HPV genome is circular, with approximately 7.9 kb double-stranded DNA, and the virus explicitly targets the basal cells of the epithelial mucosa [6]. The virus replicates as multi-copy nuclear plasmids, requiring viral DNA replication origin. The origin-binding protein, E2, and the replicative DNA helicase, E1, are the only two virus-encoded proteins that are required for DNA replication [7,8,9]. All other replication proteins are appropriated by the virus from the host cellular DNA replication machinery [10]. E2 is vital in the preinitiation complex formation to load the E1 helicase onto the replication origin [11]. The E2 protein (367 amino acids) consists of two functional domains connected by a hinge region (Figure 1) [12]. The N-terminal 201 amino acids form the transactivation domain of the E2 protein [12]. The C-terminal domain of E2 is the DNA-binding domain, consisting of 84 amino acids [13]. The E2 hinge region is an unstructured flexible linker between the transactivation and DNA-binding domains [14]. The hinge region provides space between the domains, which is necessary to counter a possible steric hindrance [14].

E2 binds to specific sequence motifs in the viral genome as determined by binding of these sequences to the DNA binding domain of E2, GST-E2 fusion protein, as well as purified full-length E2 proteins [15,16,17,18,19,20,21,22,23]. DNA replication initiation is well studied in *E. coli*, where DnaA binds to four nine bp recognition sites in the 245 bp region of the *E. coli* DNA replication origin [24,25,26,27,28,29,30,31,32,33,34,35]. Although unrelated, similar to the *E. coli* replication system, the human Alpha papillomaviruses possess four E2 binding sites in a conserved spatial arrangement in the upstream regulatory region of their genome, which constitute the origin of DNA replication [16,36,37]. Binding sites 1 and 2, separated by only three base pairs, are closest to the early promoter for *E6* and *E7* oncogenes (Figure 1). Binding sites 3 and 4 are located approximately 64 and 288 bp upstream of the *E6/E7* promoter, respectively (Figure 1). These E2 binding sites contained a conserved sequence element in many HPV types [16,19,38,39,40]. This conserved sequence is comprised of the palindromic sequence ACCGN_4_CGGT, where N is any nucleotide [20]. The central nucleotides (N_4_) are referred to as the “spacer” between the conserved half-sites of the E2 binding sites.

E2 regulates the transcription of *E6* and *E7* genes [9,21,22,23]. Binding sites 1 and 2 are involved in the transcriptional repression of viral oncogenes *E6* and *E7* [36,41,42,43]. The presence of E2 at these proximal promoter elements in the viral genome hinders the binding of cellular factors Sp1 and TFIID [36,41,42,43]. On the other hand, binding site 4 has been implicated in transcriptional activation [17]. In DNA replication, E2 binding to site 3 is required for optimal origin activation, in combination with binding to sites 1 and 2 [9,37].

Previously, we demonstrated interactions of the E2 protein with conserved ACCG and CGGT motifs in the E2 binding sites as well as with variant sequence motifs [25]. In this study, we have presented a quantitative analysis of E2 protein binding to surrounding sequence elements in its cognate binding sites to elucidate the sequence features and functional properties of the whole E2 binding sites that contribute to regulating HPV DNA replication and transcription.

## 2. Results

### 2.1. Purification of HPV-11 E2 Protein and its Structure in Solution

We have optimized the expression and purification of HPV-11 E2 protein in *E. coli,* which has enabled us to conduct mechanistic, structural, and functional studies for the full-length E2 protein. No crystal structure of the full-length E2 is currently available. Therefore, we utilized homology-based modeling to develop a model for the structure and spatial arrangement of the full-length HPV-11 E2 protein (Figure 1C).

**Figure 2:**
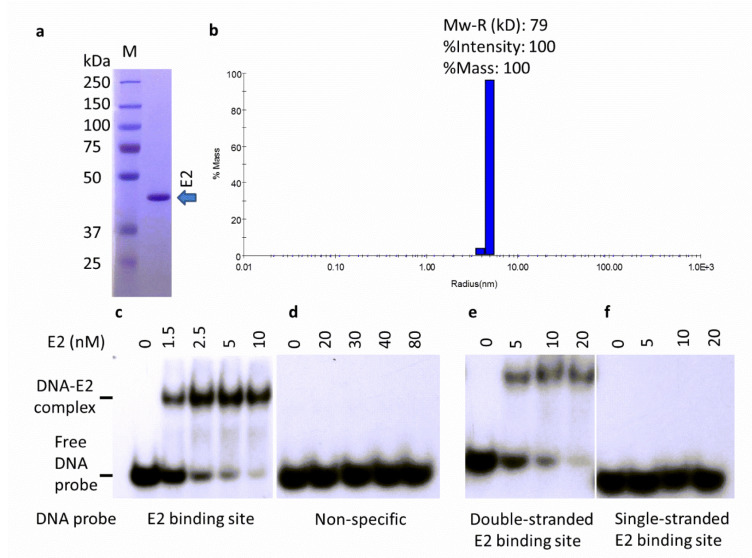
E2 protein purity, oligomeric structure, and DNA binding preference. (**a**) Coomassie Blue-stained SDS-PAGE shows purified E2 protein at a predicted size of 43 kDa. “M” represents Precision Plus Protein Standards (Bio-Rad). (**b**) Dynamic Light Scattering analysis of E2 protein sample, using three independent measurements. A representative DLS regularization graph of E2 protein showing a single mono-modal peak with a mass of 79 kDa is presented. (**c**) DNA binding by E2 protein was analyzed by EMSA using a ^32^P-labeled BS4 or (**d**) non-specific DNA probe (Table 1). (**e**) E2 protein titration of either double-stranded or (**f**) single-stranded E2 binding site. The E2 protein concentrations were as indicated.

In order to maintain solubility, E2 required high salt conditions and appeared to aggregate at lower salt concentrations. Reverse phase chromatography and histidine-affinity chromatography were ideal for its purification, as these are normally carried out at high salt. E2, purified by histidine-affinity chromatography, was analyzed by SDS-PAGE. The fractions containing E2 were pooled, subjected to reverse-phase chromatography, and eluted with a reverse salt gradient (Figure 2a). Protein digestion and sequencing confirmed the identity of purified HPV-11 E2.

The oligomeric structure of purified E2 protein in solution was determined by Dynamic Light Scattering (DLS). DLS measures the hydrodynamic radius of macromolecules in solution. The generated histogram contained a single peak, which is referred to as mono-modal size distribution, suggesting a single species population (Figure 2b). The molar mass of 79 ± 7 kDa was estimated from the measured radius of 5.05 ± 0.18 nm, indicative of a dimer (Figure 2b). Our results showed that the E2 peak comprised 100% intensity and 100% mass of the sample measured, confirming the homogeneity of the purified E2 protein.

### 2.2. E2 Protein Binds Specifically to its Double-Stranded DNA Binding Sites

The specificity of binding to four recognition sequences as well as non-specific and single-stranded DNA was quantitated by using electrophoretic mobility shift assay (EMSA). In general, ^32^P-labeled duplex oligonucleotides with or without the E2 recognition sequences were titrated with E2 protein (Table 1) (Figure 2c, Table 1). The titration of E2 with a specific oligonucleotide (E2 binding site 4) resulted in the exponential increase in a slower migrating band, indicating the formation of the E2-DNA complex (Figure 2c). However, E2 incubation with non-specific DNA showed no complex formation, even at 80 nM concentration of E2 (Figure 2d), indicating that the E2 binding to cognate DNA sequences is highly specific.

Similarly, we examined the binding of E2 to a single-stranded E2 binding site. E2 did not bind to the single-stranded form (Figure 2e,f). Together, these data suggest that E2 is bound specifically to its recognition sequence in the double-stranded structure only.

### 2.3. E2 Binds Differentially to its DNA Binding Sites

**Figure 3:**
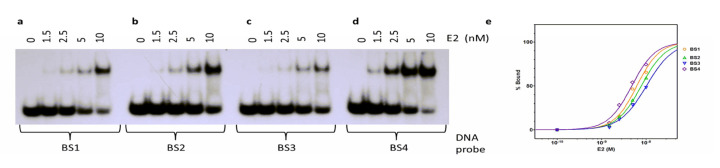
Differential binding of E2 protein to its four binding sites. (**a**–**d**) E2 protein titration to each of the four binding site probes (Table 1) labeled with ^32^P. The E2 protein concentrations were as indicated. (**e**) Results of quantitative analysis of E2 binding to BS1, BS2, BS3, and BS4 as shown in (**a**). Data represent the average of three experiments.

We explored possible differences in E2 binding affinity for its four E2 binding sites in a quantitative manner by EMSA. In this quantitative analysis, E2 protein was titrated in increasing amounts to labeled oligonucleotides containing E2 binding sites 1–4 (Figure 3). We could not go to a high enough concentration (e.g., 10 × Kd) due to the possibility of E2 precipitation in low salt conditions of EMSA, and thus all Kd values reported here should be taken as “apparent Kd.” Free and bound DNA quantities were determined using Phosphor Imager and ImageQuant TL software. The percentage of bound DNA for each binding site was plotted using GraphPad Prism 5.0 (Figure 3e). The apparent binding affinities were determined using non-linear regression analysis of the plots (% bound vs. log [E2]). The apparent dissociation constants (K_D_) were computed using a variable slope sigmoidal dose–response model in PRISM 5.0 software, and these values are shown in Table 1. These experiments were carried out in triplicate and averaged. The highest binding affinity was observed for binding site 4 (BS4), with a K_D_ of 4.8 ± 0.7 nM (Figure 3d). Intermediate binding affinity was observed with binding site 1 (BS1) and binding site 2 (BS2), with K_D_ of 6.2 ± 0.9 nM and 7.8 ± 0.7 nM, respectively (Figure 3a,b). Slightly lower binding affinity was observed with binding site 3 (BS3), corresponding to a K_D_ of 10.2 ± 1.7 nM (Figure 3c). With GST-E2 fusion protein, Thain et al. [16] found similar DNA binding patterns but with much higher Kd values, possibly due to the GST-fusion. Moskaluk and Bastia identified the binding sites of E2 using the DNA-binding domain [44]. Mok et al. [45] demonstrated DNA binding to E2BS sequences by the DNA binding domain of E2, but the Kd values were substantially higher than that reported here with the full-length protein. They also reported precipitation of the domain at lower ionic strength buffer, similar to that reported here.

**Figure 4:**
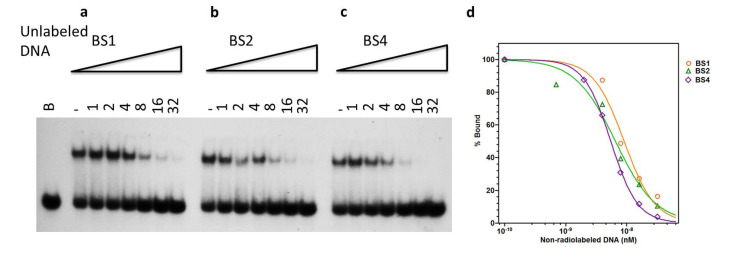
EMSA challenge assay verifies the E2 hierarchy of binding. (**a**–**c**) Challenge experiments with oligonucleotides containing individual E2 binding sites, as indicated, and titration of the E2 and ^32^P-BS1 complex formation. The E2 protein and ^32^P-BS1 concentrations remained constant. The lane that contained no E2 is marked as B. The positive control in the lane with E2 but no added competitor was represented by (-). (**d**) Quantitative analysis of challenge assay in (**a**–**c**).

In order to verify E2 binding affinities, an EMSA challenge assay was performed. In these experiments, standard amounts of E2 protein and ^32^P-labeled oligonucleotide (BS1) were titrated with an unlabeled oligonucleotide. Essentially, the addition of unlabeled oligonucleotide was expected to compete with the ^32^P-labeled oligonucleotide based on its sequence and decrease E2-DNA complex formation. As expected, BS4 was the most effective competitor, followed by BS1 and BS2 in effectiveness with K_D_ of 4.6 ± 0.4, 6.2 ± 1.4, and 6.0 ± 0.6 nM, respectively (Figure 4a–c). The plot of these data indicated a dose-dependent decrease in bound ^32^P-labeled DNA (Figure 4d). The affinities of E2 for the competing unlabeled oligonucleotides were calculated from these plots. The non-specific DNA showed no inhibition of complex formation, even at higher concentrations. Together, these data confirm the unique hierarchy and binding affinities of E2 to its binding sequences.

### 2.4. The Composition and Length of the Spacer Affected E2 Binding Affinity

The high degree of length conservation of the spacer region, between the conserved E2 half-sites, suggests physiological significance; however, it remained untested. This was the basis for our study. Oligonucleotides were designed based on wild-type E2 BS1 (ACCGAAAACGGT or ACCG(A)_4_CGGT). Spacer lengths were altered from 3 to 6 nucleotides.

**Figure 5:**
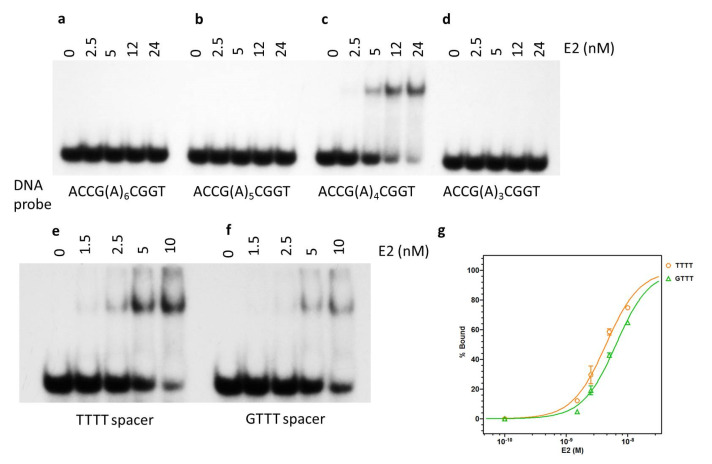
Binding of E2 to DNA binding site with altered spacer lengths and sequence. (**a**–**d**) Oligonucleotides containing E2 BS1 with varying lengths of the spacer, (A)_x_, where *X* refers to the number of adenine residues used to examine the roles of spacer length. EMSA was performed with titration of E2 protein as indicated. (**e**) Oligonucleotides contained HPV-11 E2 BS4 with either its native TTTT spacer or (**f**) spacer of BS3 (GTTT). E2 protein was titrated to each probe, with protein concentrations as indicated. (**g**) Quantitative analysis of the data derived from (**e**,**f**).

E2 protein was only able to form a DNA-protein complex with wild-type E2 BS1 containing the spacer of conserved four nucleotide length (ACCG(A)_4_CGGT) as seen in Figure 5c. No complex formation was observed with altered spacers (Figure 5a,b,d).

E2 protein bound differentially to its various binding sites, even though the core palindromic sequence remained constant (Figure 3, Table 1). All low-risk HPV genotype spacers of BS3 contain guanine, whereas the other binding sites contain AT-rich sequences primarily. The experiments reported here quantitatively analyzed the spacer sequences on binding affinity. The binding affinity for wild-type BS4 was 4.4 ± 0.4 nM, consistent with our studies mentioned previously (Figure 5e,g). An identical binding site containing a GTTT spacer yielded a binding affinity of 6.4 ± 0.6 nM (Figure 5f,g). This may partly explain the observed lowered affinity for BS3. This implies that the spacer composition does play a role in E2 binding affinity.

### 2.5. Flanking Sequences of the E2 Binding Sites Modulate E2 Binding Affinity

In order to evaluate the effects of the flanking sequences on E2 binding affinities, duplex oligonucleotides were created using a constant consensus and spacer sequence (ACCGAAAACGGT), with different 6 base pair flanking sequences obtained from the native HPV origins and presented in Table 1.

**Figure 6:**
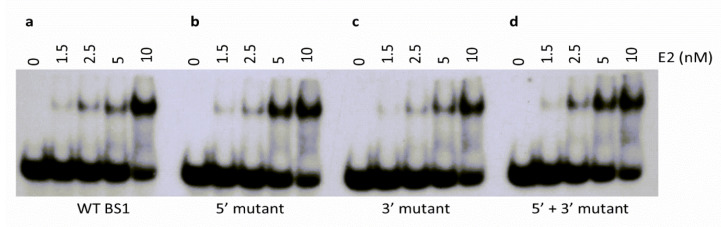
Effects of flanking sequences on DNA binding by E2 protein. The binding affinities of E2 for its four binding sites were determined by EMSA. (**a**) Core oligonucleotide sequence with native flanking sequences of E2 BS1 (WT BS1). All the other three oligonucleotides possessed alterations in the flanking sequences of BS1. (**b**) The BS1 5′ flanking sequence was replaced with the BS3 5′ flanking sequence (5′ mutant). (**c**) BS1 3′ flanking sequence was substituted with that of BS3 (3′ mutant). (**d**) The double mutant (5′ + 3′ mutant) was replaced with both the 5′ and 3′ flanking sequences from BS3. The E2 protein concentrations were as indicated.

Regardless of the sequence, E2 was able to form a DNA-protein complex with each oligonucleotide (Figure 6). When the BS1 5′s flanking sequence was replaced with the BS3 5′ flanking sequence (5′ mutant), the binding constants remained unaffected and were 6.0 ± 0.7 nM and 6.4 ± 0.6 nM, respectively (Figure 6a,b). However, the replacement of the BS1 3′ flanking sequence with that of BS3 (3′ mutant) led to a slightly higher K_D_ of 7.6 ± 0.6 nM (Figure 6c, Table 1). The double mutant (5′ + 3′ mutant) with both the 5′ and 3′ flanking sequences from BS3 bound with a K_D_ of 7.7 ± 1.7 nM (Figure 6d, Table 1). Together, our data suggested that the flanking sequences had a small but clear effect on E2 binding.

### 2.6. Modulation of DNA Binding Ability of E2 by Ionic Strength and Temperature

E2-DNA complex formation was examined as a function of NaCl concentration by EMSA. In this experiment, we used standard concentrations of E2 protein (6 nM) and ^32^P-labeled oligonucleotide (BS1). The best binding was observed at or above 25 mM. This binding remained constant even at higher salt concentrations (200 mM NaCl). Salt enhanced DNA binding by E2, and the complex was stable at high salt concentrations. The observed salt dependence suggests that DNA binding of E2 may be mostly entropy-driven or hydrophobic in nature and may be mostly devoid of ionic interactions.

**Figure 7:**
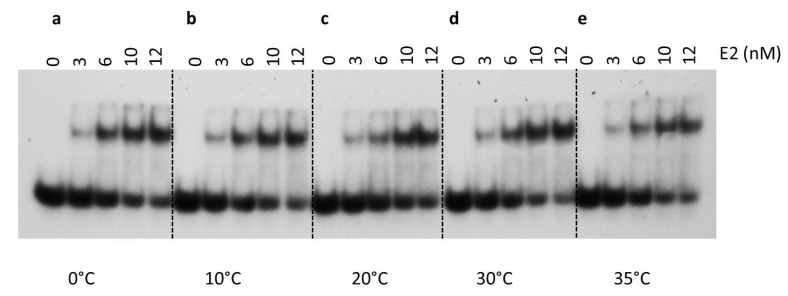
Thermodynamic parameters of E2-DNA complex formation. DNA binding by E2 was measured at indicated temperatures. E2 protein was incubated with ^32^P-labeled E2 binding site 1 with increasing concentrations of E2. Five different temperatures were analyzed: (**a**) 0 °C, (**b**) 10 °C, (**c**) 20 °C, (**d**) 30 °C, and (**e**) 35°C, as indicated.

E2 binding to BS1 was also studied at different temperatures (Figure 7a–e). Although similar binding affinities were seen at most temperatures, slightly lower binding affinity was observed at 0 °C (Figure 7a, Table 2). Thus, DNA binding was not significantly influenced by temperature and E2 was capable of binding at a wide range of temperatures. Together, results from thermodynamic and ionic strength studies suggest that the E2-DNA interaction was likely driven by hydrophobic interactions and less by ionic interactions.

### 2.7. Higher Order Complex Formation with Multiple Binding Sites

**Figure 8:**
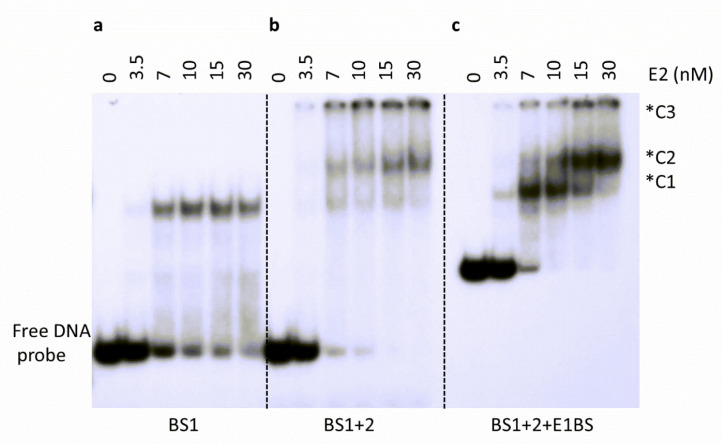
Analysis of multi-site DNA binding of E2 protein. E2 DNA binding studies with (**a**) oligonucleotides containing E2 BS1 alone (BS1), (**b**) containing both BS1 and BS2 (BS1+2), or (**c**) containing BS1, BS2, and E1 binding site (BS1+2+E1BS). EMSA was performed with increasing amounts of E2 protein as indicated above each lane. *C1 indicates a complex with one E2 dimer, *C2 indicates a complex with two E2 dimers and *C3 refers to a complex containing more than two E2 dimers. Oligonucleotide sequences are presented in Table 3.

E2 BS1 and BS2 are in close proximity in all HPV origins and are separated by a few nucleotides. Some previous studies have supported E2 DNA binding cooperativity, while others have disagreed on its involvement [46,47,48,49]. Oligonucleotides contained either one E2 binding site (BS1), or two E2 binding sites (BS1+2). The third probe (BS1+2+E1BS) used in the EMSA was generated by PCR amplification of the HPV-11 upstream regulatory region spanning HPV-11 genome nucleotide 7092-7933/1-92. This oligonucleotide incorporated BS1, BS2, and E1 binding site (E1BS). By EMSA analysis, a single E2-DNA complex (C1) was observed with one binding site and yielded a K_D_ of 5.3 ± 0.6 nM (Figure 8a), as expected. The incubation of E2 with an oligonucleotide containing two binding sites (BS1+2) resulted in a higher binding affinity of 4.2 ± 0.3 nM (Figure 8b). Notably, with the addition of this second binding site, higher-order complex formation was also observed (C2) (Figure 8b). In addition, as the formation of higher-order complexes increased, the appearance of the lower-order complexes concomitantly decreased. This pattern is more visibly evident in the oligonucleotide containing two E2 binding sites as well as an E1 binding site (BS1+2+E1BS), which was more representative of the HPV-11 origin (Figure 8c). Experiments with BS1+2 and BS1+2+E1BS yielded similar binding patterns as well as binding affinities. Together, our data suggested that the DNA binding activity of E2 may have a cooperative DNA binding component. In other words, E2 binding to the first site led to more efficient binding to the other nearby sites, as indicated by enhanced binding affinity. Understandably, the higher order complex was seen only with E2 binding to two E2 binding sites (oligonucleotides BS1+2 and BS1+2+E1BS), or two E2 dimers binding on the DNA. Another apparent feature, observed only when two E2 binding sites are present, is the formation of a third complex (C3), indicative of a much larger, multimeric E2-DNA complex indicating possible recruitment of other E2 dimers to the complex.

### 2.8. Analysis of E2-DNA Complexes by Atomic Force Microscopy (AFM)

The structures of E2-DNA complexes were visualized by using Atomic Force Microscopy (AFM), which is an ideal method for analyzing protein-DNA structures [24].

The linear DNA substrate contained the HPV-11 origin and was generated by PCR amplification of HPV-11 DNA spanning nucleotides 7076-7933/1-230. This DNA template contained all four E2 binding sites in their native spatial arrangement obtained from HPV-11 DNA and was approximately 1088 bp in length. BS4 was located in the middle of this DNA fragment, with 516 bp on one side and 560 bp on the other. The other binding sites, BS1, BS2, and BS3, were clustered at the end of the DNA fragment, located at 985 bp from one end and 169 bp from the other. The DNA was designed in this manner so that the E2-DNA complex formation will be localized at one end of the DNA fragment and not in the middle of the fragment if BS1, BS2, and BS3 form the nucleus of the complex.

**Figure 9:**
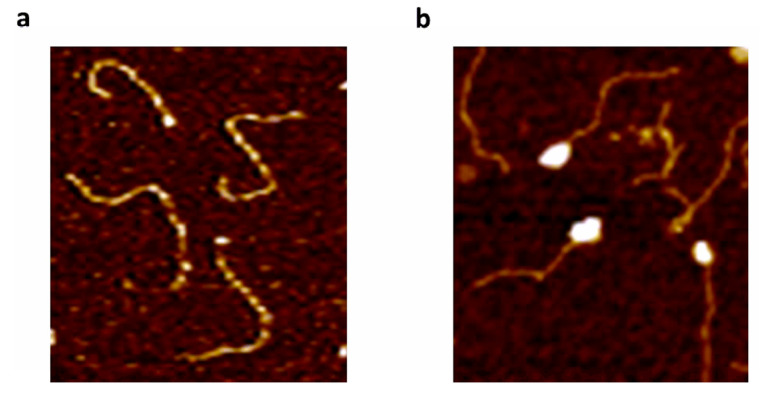
Structural analysis of E2 protein at HPV origin. The DNA substrate used in these studies was a PCR-amplified 1088 bp DNA fragment from the origin of replication (LCR) of the HPV11 genome. This DNA fragment is linear and 3.7 nm in length. (**a**) Linear DNA substrate of HPV origin was generated by PCR amplification of HPV-11 DNA spanning nucleotides 7076-7933/1-230. DNA containing all four E2 binding sites was visualized using AFM in the absence of E2 protein. (**b**) E2 protein bound to origin, observed as a white globule formation in AFM on the DNA.

E2-DNA complexes were subjected to fixation with glutaraldehyde after incubation of HPV-11 DNA fragment with E2 protein. DNA in the absence of protein was also visualized (Figure 9a). Approximately 200 molecules in each set of experiments were analyzed. E2 binding to the DNA was observed as a white globule formation along the DNA (Figure 9b). Analysis of E2-DNA complexes revealed several structures with multiple E2 bound primarily at the end of the DNA molecule (Figure 9b). The observed position of the E2 binding was consistent with the locations of the BS1, BS2, and BS3 cluster.

## 3. Discussion

HPV DNA replicates as an episomal plasmid and is initiated when dimeric E2 protein recognizes and binds to specific recognition sequences in the replication origin, creating a multiprotein-DNA initiation complex. Multiple sequence alignment of E2 proteins from different organisms demonstrated limited homology. The contributions of the large N-terminal region of E2 (~31 kDa), representing more than 75% of the protein, on DNA binding or multimer formation at the replication origin by E2 protein, could be significant.

We developed a purification scheme that allows the purification of E2 using chromatographic steps that require high salt conditions. Interestingly, DnaA, the *E. coli* origin binding protein, also requires high salt conditions to maintain solubility in an aqueous solution [50].

Based on crystallographic data derived from the transactivation domain fragment of high-risk HPV-16 E2 and the DNA-binding domain fragment of HPV-6 E2, E2 appears to exist as dimers [13,51,52,53]. Since both the transactivation and DNA-binding domain fragments form dimers in crystal, HPV E2 appears capable of forming dimers to multimers. Using light scattering measurements, we found that E2 exists as a dimer in solution at low (≤0.1 mg/mL) protein concentration (Figure 2b). Higher concentrations of the protein resulted in a mixed oligomeric population, suggesting concentration-dependent self-aggregation of E2. Incidentally, *E. coli* DnaA portrays similar self-aggregation, which may be a unique inherent property of origin-binding proteins and may be necessary for multimer formation in the replication origin [32,35,54].

### 3.1. Mechanism of E2-DNA Interaction in the HPV Origin

**Figure 10:**
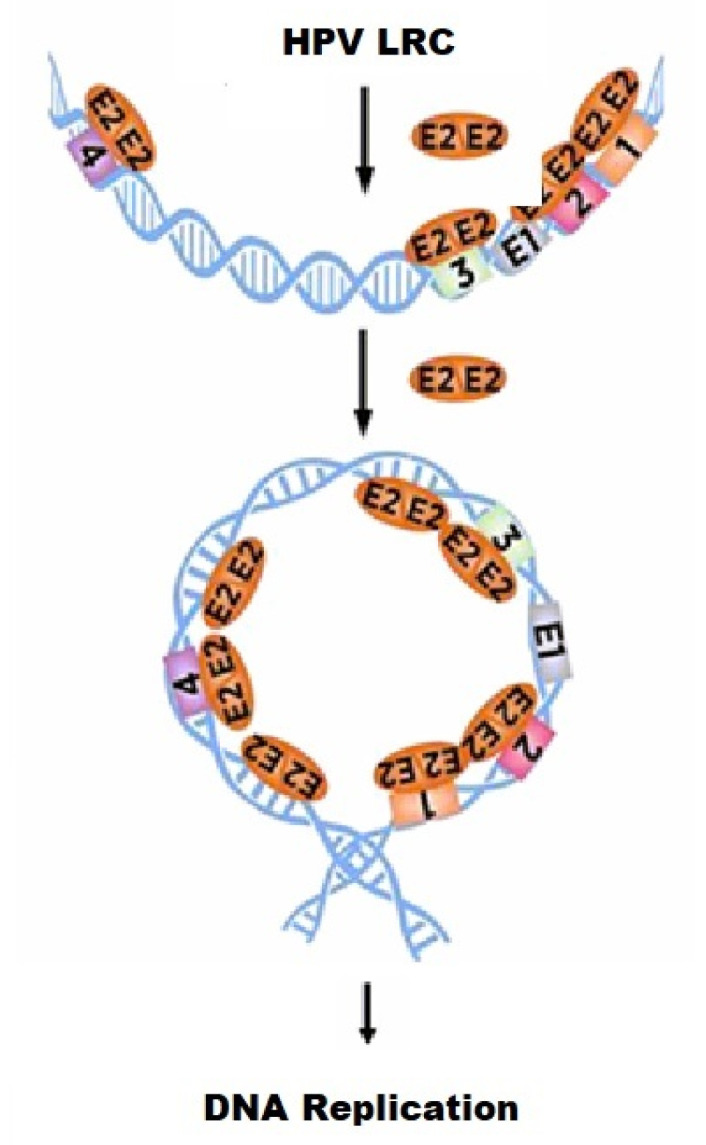
A cartoon depicting binding of multiple E2 dimers to all four E2 binding sites in the HPV LRC leading to the activation of DNA replication initiation.

E2 protein modulates HPV DNA replication and transcription through its binding to four DNA recognition sequences in the origin (Figure 1A). Recombinant full-length HPV-11 E2 protein bound all four DNA binding sites with varying affinities in EMSA analysis (Figure 3). Our results indicated that the E2 protein bound with the highest affinity to BS4 (K_D_ = 4.8 nM). The binding to all four sites was observed, and the binding affinity hierarchy was as follows: BS4 > BS1 > BS2 > BS3 with K_D_ values ranging from 4.8 to 10.2 nM (Table 1). The higher affinity binding of E2 to BS4 implies that this site will be predominantly occupied even at low concentrations of E2 protein. Subsequently, higher concentrations will allow E2 to bind BS1 and BS2, sites involved in DNA replication initiation and repression of transcription (Figure 10). These results obtained with full-length E2 protein are similar in binding pattern but greater than one order of magnitude lower than that reported by Thain et al. [16] with the GST fusion of E2 protein. Mok et al. [45] demonstrated DNA binding to E2BS sequences by the DNA binding domain of E2, but the Kd values were much higher than those found with the full-length protein. They also reported precipitation of the domain at a lower ionic strength buffer, similar to that reported here. This is consistent with previous studies demonstrating transcriptional repression with increased E2 expression [55]. Our studies demonstrated that E2 bound BS3 with the lowest affinity (K_D_ = 10.2 nM). It was earlier shown that binding to BS3 is required for DNA replication [9]. Consequently, the binding of E2 to BS3 may be the rate-limiting step in E2 activation of the HPV replication origin. Therefore, E2 binding to BS1, BS2, and finally, BS3 will lead to the activation of viral DNA replication origin (Figure 10). The order of binding affinity may be necessary for preferential and sequential occupancy of DNA binding sites by E2 dimers, especially at low E2 expression levels in the host cells [16]. Since regulation of the cellular functions by E2 is dependent on its ability to bind DNA, the binding affinities to these four sites appear critical in controlling viral processes, such as transcription and replication. Similarly, *E. coli* DnaA protein binds DNA sequences located in prokaryotic origins with varied affinities and may be a common theme amongst origin-binding proteins [24,26,30]. Our results with full-length E2 protein are strikingly different from that observed in studies carried out with the ~10 kDa HPV E2 DNA-binding domain fragment, as reported by Alexander and Phelps [40]. The binding hierarchy was diametrically opposite. The highest binding affinity was observed with BS3, intermediate binding with BS1 and BS2, and weak binding with BS4 [40]. With the full-length E2, we observed the highest binding affinity for BS4 and the lowest for BS3. A comparison of these two sets of results suggests that the 31 kDa transactivation/hinge domain plays a significant role in the modulation of the E2 DNA binding domain and its DNA binding affinity.

We also found that, because of the association properties of E2 protein, measurement of DNA binding affinity using fluorescence anisotropy, which we have used with other proteins previously, could not be utilized with E2 [24]. The self-association of the HPV E2 automatically increases the anisotropy generating aberrant K_D_ values. Therefore, EMSA analysis for determining DNA binding constants for E2 was more appropriate. Our studies in the near future include biosensor analysis of E2-DNA binding that will quantitatively determine the on (k_on_) and off (k_off_) rates of the protein DNA interaction and determine Kd (k_on_/k_off_) in an alternative means.

### 3.2. DNA Binding Is Affected by Spacer and Flanking DNA Sequences

It is well established (Table 1) that there are two sequences (ACCG and CGGT) in each E2 half-site of the palindrome, which remains highly conserved amongst the four binding sites [44]. However, our results, described above, indicated that binding affinities can vary significantly even with intact conserved sequences. Thus, we have examined whether other non-conserved sequence variations could potentially account for the differences in E2-DNA binding affinities among the four E2 binding sites. A common feature amongst various HPV genotypes was the presence of guanine in BS3 spacer sequences, whereas other E2 binding sites contained only adenine or thymine. EMSA analysis indicated that the substitution of thymine with guanine led to a decrease in the E2-DNA affinity (Figure 5f). This may partially explain the lower affinity of E2 for BS3, containing the least A/T-rich spacer of all the other sites.

Our results indicated that the flanking sequences did have an effect on E2 binding affinity in our EMSA analysis (Figure 6). When the flanking sequences of BS3 substituted those of BS1, there was a decrease in binding affinity. This may partially explain the lowered affinity of E2 for BS3. The flanking sequence of each E2 binding site is different, which may have a role in the regulation of E2 binding and affinity. Together, the results of these studies appeared to support the notion that the unique regions within and surrounding individual, naturally occurring E2 DNA binding sites promote differential E2 binding affinities, which may be a strategic feature of E2-mediated regulation.

### 3.3. Thermodynamics of E2-DNA Interaction

The nature of the E2-DNA interaction using thermodynamic analyses helped us characterize the nature of E2-DNA interaction. Our results indicated that optimum salt concentration (~25 mM NaCl) increased DNA binding, which may point to structural flexibility [56]. At higher salt concentrations (≤200 mM), binding affinity remained reasonably unaltered. The observed salt dependence suggests that DNA binding of E2 may be mostly entropy-driven or hydrophobic in nature. In order to understand the thermodynamics of E2-DNA binding interactions, E2 binding to BS1 was studied at different temperatures (Figure 7a–e). These results indicate that DNA binding was not significantly influenced by temperature. E2 is capable of binding DNA at a wide range of temperatures. Together, these studies suggest that the E2-DNA interaction is likely driven by hydrophobic interactions and less by ionic interactions.

### 3.4. Binding to DNA Containing Dual E2 Binding Sites

The spatial arrangement of E2 binding sites in the viral origin is highly conserved among papillomaviruses. The importance of this consistent layout in the context of viral regulation remains unknown. The short distance between two binding sites could permit cooperative interaction between two E2 dimers, and, subsequently, higher levels of regulation at low concentrations of full-length E2 proteins, as observed in previous studies [47,49].

### 3.5. Analysis of E2-DNA Complex at the HPV Origin by AFM

We have examined the structure of E2-DNA complexes by AFM. E2-DNA complexes formed large multiprotein-DNA structures with E2 bound mostly at the end of the DNA molecule where BS1, BS2, and BS3 were located in the 1088 bp PCR amplified HPV-11 origin DNA (Figure 9b). The observed position of the E2 protein was consistent with the locations of the BS1, BS2, and BS3 clusters (Figure 9b). This complex was, at a minimum, comprised of three E2 dimers on the DNA. Unlike a previous study using two E2 binding sites, we observed a large multi-protein complex on the DNA, without DNA looping [57]. The multi-protein structures that we observed were reminiscent of the *E. coli* replication initiator protein, DnaA, binding to its origin and forming a higher-order complex, during the initiation of DNA replication [35]. This complex structure at the origin of replication should be critical to prepare the template for priming and replication [35]. Hence, the oligomerization of full-length origin-binding proteins may be a necessary pre-requisite for multi-protein complex formation and the activation of the replication origin as depicted in Figure 10.

## 4. Materials and Methods

### 4.1. Reagents

All chemicals were ACS reagent grade and from Fisher Chemical Company, Pittsburgh, PA. Synthetic oligonucleotides were obtained from Millipore Sigma, St. Louis, MO, USA. Sequences are as presented in Table 1 and Table 3. Recombinant plasmids were sequenced by Eurofins MWG Operon LLC, Louisville, KY, USA.

### 4.2. Buffers

Buffer A contained 25 mM Tris-HCl (pH 8.0), 0.1 M NaCl, and 10% sucrose. Buffer B comprised 20 mM Tris-HCl (pH 8.0), 500 mM NaCl, and 2 mM DTT. Buffer B_X_ refers to buffer B containing *X* mM imidazole.

### 4.3. Full-Length Protein Structure Modeling

The structure of the full-length HPV-11 E2 protein was modeled using Robetta protein structure prediction software (http://www.robetta.org/) accessed on 3 March 2023, at the University of Washington, Seattle, WA. Robetta methodology uses a combination of Rosetta homology modeling software and ab initio fragment assembly with Ginzu domain prediction software that allows the modeling of full-length protein with partial or no available crystal structure(s). This methodology was chosen for creating the three-dimensional model as crystal structures of the individual domains of E2 are available, but none is available for full-length E2 protein.

### 4.4. Cloning, Expression, and Purification of E2

The HPV-11 *E2* gene was PCR amplified from purified plasmid HPV-11 DNA, GenBank number M14119 (ATCC, Rockland, MA, USA). The PCR product was cloned into a T7 expression vector, pET28a, engineered to have an N-terminal histidine tag (EMD Millipore, Billerica, MA, USA), using standard recombinant DNA technology [58]. The validity of the cloned *E2* gene, in the pET28a-E2 plasmid, was verified by DNA sequencing. *E. coli* Lemo21 (DE3) strain (New England Biolabs, Ipswich, MA, USA) was then transformed with the pET28a-E2 plasmid for subsequent expression of the protein. *E. coli* cells were grown with 1 mM L-rhamnose and shaken at 37 °C to OD_600_ of 0.4. Isopropyl-β-D-thio-galactopyranoside (IPTG) was then added to a final concentration of 0.4 mM, and incubation at 37 °C was continued for 16 h. The cells were harvested by centrifugation and resuspended in Buffer A containing 1 µg/mL of each protease inhibitor, including pepstatin, leupeptin, chymostatin, and aprotinin. Induced cells were extracted with the addition of lysozyme following standard protocol [59]. The extract was loaded onto cOmplete His-tag purification resin (Roche, Pleasanton, CA, USA), and equilibrated with buffer B_10_. The column was washed thoroughly with buffer B_25_, followed by a brief wash with buffer B_50_. The protein was eluted using buffer B_200_ containing 10% glycerol. Fractions containing E2 were identified by SDS-PAGE. These fractions were pooled and subjected to reverse-phase chromatography. The protein was eluted using a reverse gradient from high salt (2 M NaCl) to lower salt (0.5 M NaCl). E2 eluted near 0.5 M NaCl concentration in the fully soluble form. Protein homogeneity was determined by SDS-PAGE (Figure 2a) and verified by mass spectrometry conducted at the University of Florida (Gainesville, FL, USA) Department of Chemistry Mass spectrometry services.

### 4.5. Mass Spectrometry

Purified HPV-11 E2 protein (2 µg) was loaded onto a 10% polyacrylamide gel. The gel was fixed for 15 min following adequate gel separation. Fixation was followed by a rinse cycle and subsequently stained with Coomassie Blue. The SDS-PAGE gel was destained with a fixing solution. The clearly visible band was extracted from the rest of the gel. Protein sequencing and analysis were performed by the University of Florida (Gainesville, FL, USA) Department of Chemistry Mass spectrometry services.

### 4.6. Dynamic Light Scattering of Purified E2 Protein

Dynamic Light Scattering experiments were performed using DynaPro NanoStar instrument and DYNAMICS 7.1 software (Wyatt Technology, Santa Barbara, CA). Sample parameters were set, including solvent as 10% glycerol. Mw-R model used was “branched polymers.” The measurement time period was 400 s, consisting of 20 acquisitions, at 20 s each. Three separate measurements were performed.

### 4.7. Electrophoretic Mobility Shift Assay (EMSA)

Synthetic oligonucleotides were used for end-labeling reactions with T4 polynucleotide kinase and (γ-^32^P) ATP. DNA probes were purified by Bio-Gel P-6 column (Bio-Rad, Hercules, CA, USA).

The binding reactions were carried out in a 10-µL final volume containing the indicated amount of protein and 70 pmol of γ ^32^P-labeled DNA probe in 10 mM HEPES (pH 7.9), 4 mM MgCl_2_, 10% glycerol, 5 mM dithiothreitol, 0.2 mM EDTA, 0.1 mg/mL bovine serum albumin, 10 ng/µL poly(dI-dC), and 50 mM NaCl. The binding reactions were allowed to proceed for 10 min at 30 °C instead of 37 °C to minimize the effect of any possible trace nuclease, DNase, or protease contaminants. The mixtures were loaded on 5% polyacrylamide gel in 0.5X TBE and run at 170 V for 45 min at 4 °C, except for the multiple E2 binding assay, which was run for 2 h at 170 V. Following electrophoresis, the gels were dried and exposed to X-ray films. Quantification was accomplished by Phosphor Imager analysis using Typhoon 9410 Variable Mode Imager (GE Healthcare Life Sciences, Santa Cruz, CA, USA). In competition experiments, reactions were performed as indicated above, with the addition of non-radiolabeled/competitor oligonucleotides in increasing amounts. The competitor DNA was added simultaneously with the labeled probe. Each experiment was quantified independently by Phosphor Imager analysis.

### 4.8. Calculation of Equilibrium Binding Constants

The radioactive (^32^P) labeled free probe and the complex in each lane after EMSA was quantified by phosphor Imager analysis using Typhoon 9410 Variable Mode Imager (GE Healthcare Life Sciences) as indicated in Section 4.7. The integrity of the quantitation of the bands was controlled by the length of the exposure. The % bound probe (B) was determined by the following equation: B = 100 (1 − y/x)

Non-linear regression analysis of the fractional occupancy (bound probe vs. log (E2)) was carried out using Prism 6.0 software (GraphPad Software Inc, San Diego, CA, USA) utilizing a variable slope sigmoidal dose–response model.

### 4.9. Cloning of HPV-11 Origin for Atomic Force Microscopy of E2-DNA Complexes

The HPV-11 origin (GenBank number M14119 from ATCC) could not be amplified directly from purified plasmid HPV-11 DNA, due to the interruption of the HPV-11 origin by pBR322 vector DNA. As a result, the HPV-11 DNA was excised from the vector and ligated into circles. Briefly, the HPV-11 plasmid was digested with restriction enzyme BamH1 and separated on a 1% agarose gel. The correct DNA band was excised from the gel and purified using Gene Clean Kit (MP Biomedicals, Santa Ana, CA, USA), followed by self-ligation using Clonables Ligation Premix (EMD Millipore, Billerica, MA, USA). Following ligation of the DNA, the HPV-11 origin containing all four E2BSs was generated Afrom the circular DNA by PCR using primers 5′-CTCCTCGGATCCTGGAGGACTGGAACTTTGGT-3′ and 5′-TCTTCTAAGCTTTATCTC TGCGGTGGTCAGTG-3′. PCR product was purified using GeneJet PCR Purification Kit. The correct DNA band from the purified PCR product was separated on a 0.8% agarose gel, excised from the gel, and purified. Using standard recombinant DNA technology, the PCR product was cloned into an M13KS vector for sequencing purposes. The plasmid was fully sequenced prior to further studies.

### 4.10. Binding Reactions for AFM

The binding reactions were carried out in a 20-µL final volume containing 100 ng of linear DNA and 150 ng of E2 protein in 20 mM HEPES (pH 7.9) and 2 mM ATP, modified from a previously published method [48]. The binding reactions were allowed to proceed for 20 min at 37 °C and then treated with glutaraldehyde for 5 min at room temperature. E2-DNA complexes were brought to a final volume of 50 µL with 20 mM HEPES (pH 7.9). These complexes were then separated by gel filtration through 0.5 mL columns of 6% crosslinked agarose beads (Agarose Bead Technologies Inc., Miami, FL, USA) equilibrated with 20 mM HEPES (pH 7.9). Atomic Force Microscopy (AFM) imaging was performed at Delaware Biotechnology Institute (University of Delaware, Newark, DE, USA) using a multimode AFM in peak force tapping mode. The E2-DNA complex solution (5 µL) and 5 µL 2M NiCl_2_ in HEPES was deposited on freshly cleaved mica and incubated at room temperature for 5 min for binding, followed by four rinses with deionized water. The samples were then allowed to air dry and positioned for imaging. The AFM images were analyzed by using NanoScope Analysis software v1.8 (Bruker, Billerica, MA, USA).

## 5. Conclusions

- E2 binding sites have conserved sequences that result in efficient DNA recognition and high affinity binding.
- Four-nucleotide spacer sequence as well as flanking DNA sequences around individual E2 DNA binding site modulated DNA binding affinities.
- Full-length E2 protein was a dimer in solution only at low concentrations and formed larger oligomers at higher concentrations.
- The E2-DNA interaction is thermodynamically driven primarily by hydrophobic interactions.
- AFM analysis revealed that the association of E2 with the HPV origin resulted in multimer formation, a prerequisite for the initiation of loop formation.

## Figures and Tables

**Table 1:** Sequences for various oligonucleotides used as E2 binding sites and the associated dissociation constants are presented. Sequences for only one strand of each duplex oligonucleotide is shown. E2 consensus sequences are underlined. Mutant flanking sequences are bolded. Each K_D_ was calculated and averaged from the experiment performed in triplicate. K_D_ values represent the average of three independent measurements. Standard deviations were incorporated into K_D,_ accordingly. * ND stands for “not determined”.

Oligonucleotide	Sequence	Kd [nM]
Non-specific	TGGCGGACCCCACCCAGCCCCACGCGCA	ND *
BS1	GGTTCA ACCGAAAACGGT TATATG	6.2 ± 0.9
BS2	GGAGGG ACCGAAAACGGT TCAACC	7.8 ± 0.7
BS3	CCTGCA ACCGGTTTCGGT TACCCA	10.2 ± 1.7
BS4	CTTGCA ACCGTTTTCGGT TGCCCT	4.8 ± 0.7
BS1 5′ mutant	5′ **CCTGCA** ACCGAAAACGGT TATATG 3′	6.4 ± 0.6
BS1 3′ mutant	5′ GGTTCA ACCGAAAACGGT **TACCCA** 3′	7.6 ± 0.6
BS1 5′ + 3′ mutant	5′ **CCTGCA** ACCGAAAACGGT **TACCCA** 3′	7.7 ± 1.7

**Table 2:** Thermodynamic analysis of binding affinities for E2 protein. Each K_D_ was calculated for the temperatures as indicated.

Temperature	K_D_ (M)
0 °C	7.3 ± 0.7 × 10^−9^
10 °C	5.5 ± 1.5 × 10^−9^
20 °C	6.2 ± 1.4 × 10^−9^
30 °C	5.3 ± 0.4 × 10^−9^
35 °C	5.7 ± 1.1 × 10^−9^

**Table 3:** Sequences of oligonucleotides utilized in multi-site DNA binding assays. Sequences for only one strand of each duplex oligonucleotide are shown. E2 binding sites (BS1 and BS2) are underlined and bolded.

Oligonucleotide	Sequence
BS1 (50 bp)	GGAGGGATTGAAAACTTTTCAACCGAAAACGGTTATATATA AACCAGCCC
BS1+2 (50 bp)	GGCGGGACCGAAAACGGTTCAACCGAAAACGGTTATATATA AACCAGCCC
BS1+2+E1BS(124 bp)	GTAACCCACACCCTACATATTTCCTTCTTATACTTAATAACAA TCTTATTTAAAAAAGAGGAGGGACCGAAAACGGTTCAACCG AAAACGGTTATATATAAACCAGCCCAAAAAATTAGCAGA

## Data Availability

Data presented here will be freely available.
